# Structural Diversity and Anti-Diabetic Potential of Flavonoids and Phenolic Compounds in *Eriobotrya japonica* Leaves

**DOI:** 10.3390/molecules30030736

**Published:** 2025-02-06

**Authors:** Min Hee Kim, Sang Won Yeon, Se Hwan Ryu, Hak Hyun Lee, Ayman Turk, So Yeong Jeong, Young Jun Kim, Ki Yong Lee, Bang Yeon Hwang, Mi Kyeong Lee

**Affiliations:** 1 College of Pharmacy, Chungbuk National University, Cheongju 28160, Republic of Korea; smflaqh7412@chungbuk.ac.kr (M.H.K.); sangwon1352@chungbuk.ac.kr (S.W.Y.); sehwan0188@chungbuk.ac.kr (S.H.R.); leehakhyun1997@chungbuk.ac.kr (H.H.L.); aymanturk@chungbuk.ac.kr (A.T.); wjdthdud2912@chungbuk.ac.kr (S.Y.J.); byhwang@chungbuk.ac.kr (B.Y.H.); 2 College of Pharmacy, Korea University, Sejong 47236, Republic of Korea; yjkim22@korea.ac.kr (Y.J.K.); kylee11@korea.ac.kr (K.Y.L.)

**Keywords:** *Eriobotrya japonica*, flavonoids, phenolics, coumaroyl flavonoids, α-glucosidase, loquat

## Abstract

*Eriobotrya japonica* (Thunb.) Lindl., commonly known as loquat, is a plant belonging to the Rosaceae family. While its fruit is widely consumed as food and used in traditional medicine, research on other parts of the plant remains insufficient. Therefore, the chemical constituents and biological activities of its leaves were investigated. Phytochemical analysis of *E. japonica* leaves identified 30 compounds, including flavonoids, phenolics, and megastigmanes. The flavonoids isolated from the leaves include flavones, flavans, flavolignans, flavonoid glycosides, and coumaroyl flavonoid glycosides. Coumaroyl flavonoid rhamnosides were characteristically present in *E. japonica* leaves, and the configurations of coumaric acids, as well as the binding position to the rhamnose in each compound, were identified through detailed NMR analysis. Notably, three of them were isolated from this plant for the first time. Phenolic compounds were found to be present as conjugates with organic acids, such as quinic acid, shikimic acid, and glucose. Flavonoid and phenolic compounds demonstrated significant antioxidant and α-glucosidase inhibitory effects, whereas megastigmanes showed little activity. Notably, coumaroyl flavonoid rhamnosides, which consist of flavonoids combined with the phenolic acid, coumaric acid, exhibited excellent anti-diabetic effects. Further molecular docking analysis confirmed that these compounds effectively bind to the α-glucosidase enzyme. In conclusion, the present study identified flavonoid and phenolic components with various structures in *E. japonica* leaves and clarified their anti-diabetic and antioxidant effects. These findings support the beneficial potential of *E. japonica* leaves for the treatment and/or prevention of metabolic diseases.

## 1. Introduction

*Eriobotrya japonica* (Thunb.) Lindl., commonly known as loquat, is a species of flowering plant in the family Rosaceae. It is distributed across Asia including China, Japan and Korea as well as in the Mediterranean. All parts of *E. japonica*, including its leaves, fruit, root, and stem, have been known to contain diverse bioactive compounds with significant potential benefits [1,2,3]. Among them, the fruits of *E. japonica* are primarily consumed fresh or used in making jams and alcoholic beverages. Consequently, its leaves are often produced as byproducts of fruit cultivation. It is also known to be rich in bioactive constituents such as triterpenoids, flavonoids, phenolic, and polysaccharides, which exhibit antioxidant, anti-diabetic and anti-cancer activities [4,5,6].

Diabetes is one of the most common metabolic diseases worldwide, posing a threat to human life. It is characterized by chronic high blood sugar levels primarily due to the problems with insulin. Insulin resistance, insulin deficiency, and excessive glucose production are major causes of diabetes. Lifestyle and environmental factors also trigger its development. Sustained hyperglycemia, if not properly managed, can lead to severe health complications such as cardiovascular diseases, nephropathy, neuropathy, and retinopathy [7,8,9].

Oxidative stress is caused by an imbalance between reactive oxygen species (ROS) production and the body’s defense ability. Persistent oxidative stress resulting from excessive production of ROS can lead to severe conditions such as cancer, inflammation, and metabolic diseases including diabetes. Pancreatic β-cells, which produce insulin, are particularly susceptible to oxidative stress due to their low antioxidant defense system. Damaged β-cells fail to produce sufficient insulin, making blood sugar regulation difficult. Additionally, oxidative stress also interferes with the action of insulin, leading to insulin resistance. In diabetes, hyperglycemia exacerbates ROS production through mitochondrial dysfunction and glucose autoxidation. Thus, oxidative stress worsens diabetes by contributing to insulin resistance and chronic complications, and diabetes triggers ROS production, which promotes progression to more severe states [10,11,12].

Research on treatments for diabetes and symptom alleviation is actively ongoing [13,14]. Among therapeutic targets, lowering blood sugar levels, particularly postprandial levels, is critical. α-Glucosidase is an enzyme that breaks down complex carbohydrates into simple sugars in the small intestine. Inhibitors of this enzyme slow down the release of glucose into the bloodstream, preventing a sharp rise in blood glucose levels. α-Glucosidase inhibitors are effective in controlling postprandial blood sugar levels and several α-glucosidase inhibitors like acarbose and voglibose are used to treat carbohydrate-related disorders [15,16]. Antioxidants are also utilized to mitigate diabetes by enhancing insulin action and promoting β-cell survival through the inhibition of ROS. They also alleviate complications caused by diabetes [17,18]. Therefore, combining α-glucosidase inhibitors and antioxidants can provide effective diabetes treatment by controlling postprandial blood sugar and reducing complications.

Natural products have been extensively studied as potential therapeutic agents for diabetes due to their bioactive compounds with diverse pharmacological activities, including antioxidant, anti-hyperglycemic, and anti-inflammatory properties. Natural products with α-glucosidase inhibitory activity are considered crucial for managing and preventing diabetes by regulating blood glucose levels [19,20]. Plant-derived natural antioxidants, particularly polyphenols, are highly beneficial for combating oxidative stress and metabolic diseases [21,22]. Thus, natural products play a significant role in diabetes prevention and treatment by controlling blood glucose and protecting against complications.

Traditionally, *E. japonica* has been used for the treatment of diabetes, infections, and inflammation. Research on its anti-diabetic effects has been conducted, and the effects of polysaccharides and flavonoids have been reported [4,5]. However, the available evidence remains insufficient. In this study, the potential of *E. japonica* leaves in diabetes management was investigated by measuring both α-glucosidase inhibitory and antioxidant activities. Thirty compounds were isolated from *E. japonica* leaves and evaluated for their α-glucosidase inhibitory and antioxidant activities.

## 2. Results and Discussion

### 2.1. Biological Activity and Contents of Flavonoids and Phenolic Compounds

The anti-diabetic and antioxidant effects of the extracts and fractions of *E. japonica* leaves were evaluated. The anti-diabetic effect was determined by measuring the inhibitory activity of glucosidase, and the antioxidant effect was determined using DPPH radical scavenging activity.

**Figure 1:**
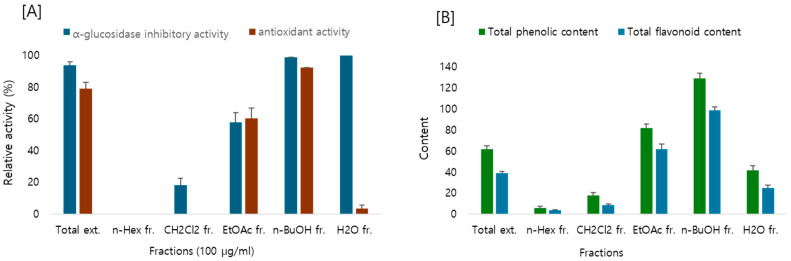
(**A**) α-Glucosidase inhibitory and DPPH radical scavenging activity and (**B**) total flavonoid and phenolic content of *E. japonica* leaves. Acarbose and ascorbic acid were used as positive controls.

As shown in Figure 1, total extract of *E. japonica* showed strong antioxidant and α-glucosidase inhibitory effects. Among the fractions tested, EtOAc and *n*-BuOH fractions showed both antioxidant and α-glucosidase inhibitory activities, whereas H_2_O fraction exhibited only α-glucosidase inhibitory activities.

Previous studies have identified terpenoids and phenolic compounds as major components of this plant. Among these, flavonoids are particularly known for their antioxidant and anti-diabetic properties. HPLC analysis of *E. japonica* extracts suggested the presence of flavonoids. Based on these findings, the total phenolic and flavonoid contents of the EtOAc and *n*-BuOH fractions, which showed excellent efficacy, were quantified. Consistent with the efficacy results, the EtOAc and *n*-BuOH fractions contained high levels of phenolics and flavonoids.

Thus, it was hypothesized that the EtOAc and *n*-BuOH fractions contain bioactive components, including phenolic compounds and flavonoids. Subsequently, efforts were made to isolate these substances for further study.

### 2.2. Isolation and Characterization of the Constituents of E. japonica

**Figure 2:**
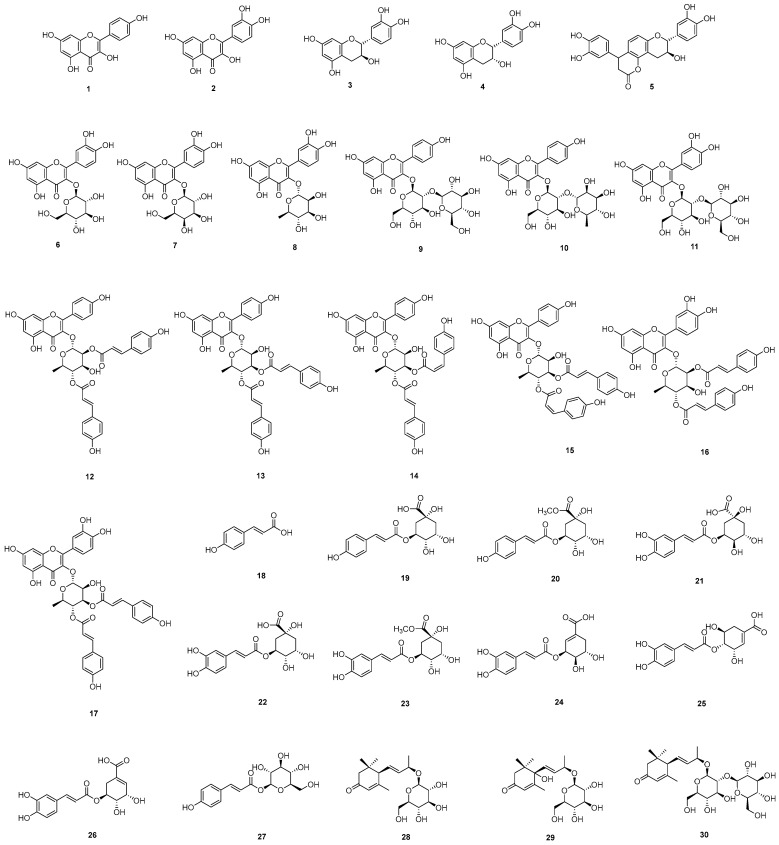
Chemical structures of compounds **1**–**30** from *E. japonica*.

Using various chromatography methods, thirty (**1**–**30**) compounds were isolated from *E. japonica* leaves (Figure 2). The structures of the isolated compounds were identified as 17 flavonoid derivatives kaempferol (**1**), quercetin (**2**), catechin (**3**), epicatechin (**4**), cinchonain (**5**), quercetin-3-*O*-β-glucoside (**6**), quercetin-3-*O*-β-galactoside (**7**), quercetin-3-*O*-α-rhamnoside (**8**), kaempferol-3-*O*-β-sophoroside (**9**), kaempferol-3-neohesperidoside (**10**), quercetin-3-*O*-sophoroside (**11**), kaempferol-3-*O*-α-rhamnopyranoside 2″,4″-di-*E-p*-coumaroic acid ester (**12**), kaempferol 3-*O*-α-(3″E,4″E di-*p*-coumaroyl)-rhamnoside (**13**), kaempferol-3-*O*-α-rhamnoside 3″-*Z*,4″-*E*-di-*p*-coumaroic acid ester (**14**), kaempferol-3-*O*-α-rhamnoside 3″-*E*,4″-*Z*-di-coumaroic acid ester (**15**), quercetin 3-*O*-(2″,4″di-*E*-*p*-coumaroyl)-α-rhamnoside (**16**), quercetin 3-*O*-(3″,″di-*E-p*-coumaroyl)-α-rhamnoside (**17**), 10 phenolic compounds, *trans**-**p*-coumaric acid (**18**), 5-*O*-coumaroylquinic acid (**19**), 5-*O*-coumaroylquinic acid methyl ester (**20**), 3-*O*-caffoylquinic acid (**21**), 5-*O*-caffeoylquinic acid (**22**), 5-*O*-caffeoylquinic acid methyl ester (**23**), 3-*O*-caffeoylshikimic acid (**24**), 4-*O*-caffeoylshikimic acid (**25**), 5-*O*-caffeoylshikimic acid (**26**), 1-*O-p*-coumaroyl-β-glucoside (**27**) and 3 megastigmanes, 3-oxo-α-ionol β-glucoside (**28**), roseoside (**29**), as well as 3-oxo-α-ionol-9-*O*-β-glucosyl(1→2)-β-glucoside (**30**) via analysis of their physical data and comparison with literature values [23,24,25,26,27,28,29,30,31,32,33,34,35,36,37,38,39,40,41,42,43].

The flavonoid derivatives can be further categorized into four subclasses: two flavonoids (**1**, **2**), two flavans (**3**, **4**), one flavolignan (**5**), six flavonoid glycosides (**6**–**11**), and six coumaroyl flavonoid glycosides (**12**–**17**). The coumaroyl flavonoid glycosides are characterized by structures in which rhamnose and two coumaric acids are attached to the flavone skeleton. These compounds differ in the configuration of the double bond in the coumaric acid and the position where the coumaric acid is bonded to the rhamnosyl moiety. These structural differences can be elucidated using NMR spectroscopy.

**Figure 3:**
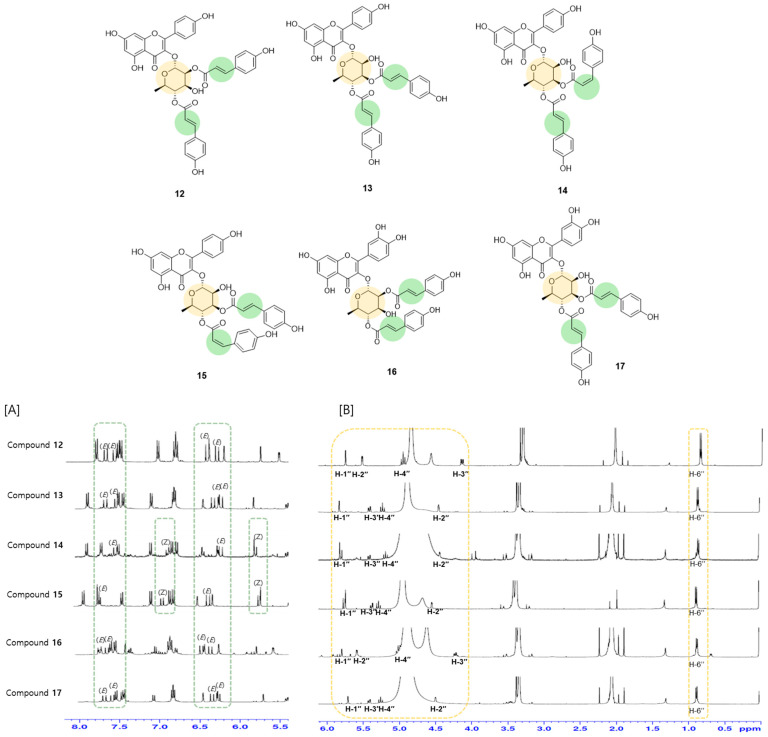
Comparison of NMR spectrum for compounds **12**–**17**: (**A**) rhamnosyl protons in orange and (**B**) olefinic double bonds in green.

The configuration of the coumaric-acid double bond is either *E* (trans) or *Z* (cis), which is determined by the *J* values of 16.0 Hz and 12.8 Hz, respectively. Compounds **12**, **13**, **16**, and **17** contain exclusively *trans-p*-coumaric acid, while compounds **14** and **15** contain *cis-p*-coumaric acid. This conclusion is based on the NMR signals observed at [δ_H_ 6.85 (1H, d, *J* = 12.8 Hz, H-7‴), 5.76 (1H, d, *J* = 12.8 Hz, H-8‴)] and [δ_H_ 6.84 (1H, d, *J* = 12.8 Hz, H-7′′′′), 5.65 (1H, d, *J* = 12.8 Hz, H-8′′′′)], which show relatively small coupling constants and upfield chemical shifts (Figure 3A).

In compounds **12**–**17**, the two coumaroyl moieties are attached to the rhamnose unit. The binding positions of the *p*-coumaric acid can be deduced from the chemical shifts in the rhamnosyl protons. The binding of *p*-coumaric acid to the rhamnosyl moiety induces a downfield shift in the rhamnosyl proton signals. As shown in Figure 3B, the 2″-rhamnosyl protons exhibit a downfield shift to δ_H_ 5.54 in compounds **12** and **16,** and the 3″-rhamnosyl protons are shifted downfield to δ_H_ 5.37 in compounds **13**, **14**, **15**, and **17**.

The phenolic compounds include a simple phenolic (**18**), four phenolic-quinic acid conjugates (**19**–**22**), three phenolic-shikimic acid conjugates (**23**–**26**), and phenolic glycoside (**27**).

As shown in our present results, loquat leaves contain various phenolic and flavonoid components. The flavonoids have diverse skeletons and exist as aglycones or glycosides with different types and numbers of sugars. Flavolignan, in which a phenolic compound is bound to flavonoids, is also present. In addition, coumaroyl flavonoid rhanmosides, in phenolic acids bound to glycosyl moiety of flavonoid glycosides, are also characteristically observed. Notably, compounds **15**–**17** were first reported from this plant. Loquat leaves also possess diverse phenolic compounds conjugated with quinic acid or shikimic acid as well as simple structures and glycosides. In addition, structural diversity was also achieved through the binding of hydroxyl and methoxyl groups. In addition to phenolic compounds and flavonoids, it contained megastigmane compounds, which proved the phytochemical diversity in loquat leaves.

### 2.3. Evaluation of Antioxidant and α-Glucosidase Inhibitory Activity

The biological activity of the isolated compounds was evaluated through DPPH radical scavenging and α-glucosidase inhibitory assays. The isolated compounds demonstrated significant antioxidant and α-glucosidase inhibitory activities, although the effects varied depending on their structures (Table 1).

As described above, the compounds isolated from loquat leaves in this study include phenolic, flavonoid, and megastigmane. Among them, phenolic and flavonoid compounds exhibited strong activities, whereas megastigmanes showed little effects. The flavonoids isolated in this study comprised flavones, flavans, flavolignans, flavonoid glycosides, and coumaroyl flavonoid glycosides. Most flavonoids exhibited strong α-glucosidase inhibitory activity, but the efficacy was reduced when sugars were bound to the flavonoid aglycones. Phenolic compounds also displayed excellent α-glucosidase inhibitory activity. Particularly, coumaroyl flavonoids, in which coumaric acids—phenolic compounds—were conjugated with flavonoids, showed strong α-glucosidase inhibitory effects.

The activity (%) is as follows: [100 − 100 × (absorbance of sample with enzyme − absorbance of sample only)/(absorbance of enzyme − blank)]

Isolated compounds also exhibited antioxidant activity. Compounds with a 3,4-dihydroxy group in the B ring demonstrated particularly high efficacy, as consistent with previous studies [44].

**Figure 4:**
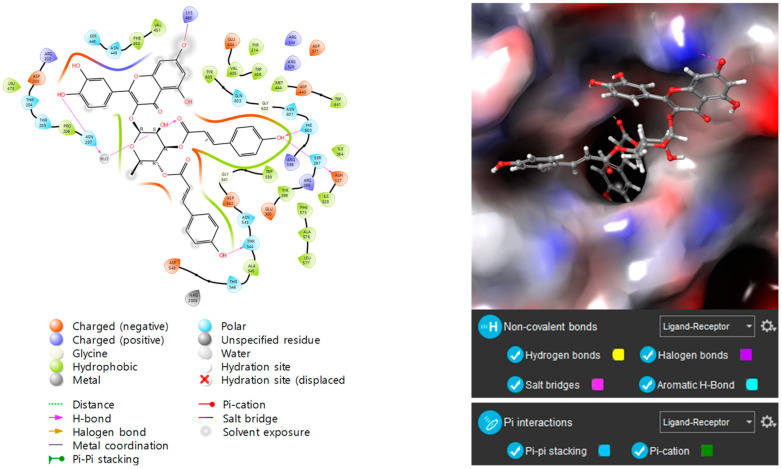
Docking picture of compound **17** to α-glucosidase.

As coumaroyl flavonoid glycosides (**12**–**17**) were found to be excellent α-glucosidase inhibitors, molecular docking analysis was performed targeting human maltase-glucoamylase (PDB ID: 2QMJ, N-terminal subunit) to further investigate the mechanism. Consistent with the experimental results, coumaroyl flavonoid glycosides (**12**–**17**) achieved high docking scores (See Supplementary Material). As illustrated in Figure 4, compound **17** exhibited the highest docking score, forming multiple bonds with the enzyme.

In conclusion, thirty compounds, including flavonoids, phenolic compounds, and megastigmanes, were isolated from *E. japonica* leaves. These compounds exhibited diverse substituents, which contributed to structural diversity and also influenced their biological effects.

The constituents of loquat leaves have been reported to include triterpenes, flavonoids, sesquiterpenes, megastigmanes, phenylpropanoids, and polysaccharides [4,5,6]. This study aimed to characterize the active components through chromatographic purification of the EtOAc and *n*-BuOH fractions, which showed α-glucosidase inhibitory and antioxidant activities. As a result, flavonoids and phenolic substances were identified as the major active constituents.

Various flavonoids with different structures were isolated, including flavones, flavans, flavolignans, flavonoid glycosides, and coumaroyl flavonoid glycosides, from loquat leaves. Additionally, several phenolic compounds conjugated with organic acids such as quinic acid, shikimic acid, and glucose were identified. Notably, six coumaroyl flavonoid rhamnosides were found to be unique to loquat leaves, with three of them being isolated from this plant for the first time.

The isolated compounds were tested for α-glucosidase inhibitory and antioxidant activities, suggesting their potential as anti-diabetic agents. Previous studies have reported the anti-diabetic efficacy of loquat leaves, including hypoglycemic and anti-obesity effects in various in vitro and in vivo models [5,45,46]. Triterpenes and polysaccharides have been reported as the active components [47,48,49]. Here, the α-glucosidase inhibitory and antioxidant effects of flavonoids and phenolic compounds from loquat leaves were demonstrated. While flavonoids and phenolic compounds are widely distributed in plants and known for various activities including anti-diabetic effects [28,32,44], their activities in loquat leaves have not been systematically studied. Here, we explored the structure–activity relationship by comparing the biological effects of the isolated compounds with diverse structures. Notably, coumaroyl flavonoid rhamnosides, including the newly identified compounds from this plant, exhibited excellent anti-diabetic effects. Furthermore, molecular docking analysis confirmed the effective binding of these compounds to α-glucosidase enzymes, providing additional insight into their mechanism of action.

This study indicates that *E. japonica* leaves are rich in flavonoids and phenolic compounds, which exhibit α-glucosidase inhibitory and antioxidant activities. These findings support the anti-diabetic and antioxidant potential of *E. japonica* leaves, and additional studies on toxicity, efficacy, and clinical trials are necessary to develop an anti-diabetic treatment.

## 3. Materials and Methods

### 3.1. Plant Material

The leaves of *E. japonica* which were cultivated in the Chonnam Province of Korea were purchased from an herbal market (Jecheon, Republic of Korea). The specimen was identified by the herbarium of the College of Pharmacy, where voucher specimens (CBNU2016-LLF) were deposited.

### 3.2. General Experimental Procedure

A Bruker DRX 400 or 500 MHz spectrometer (Bruker-Biospin, Karlsruhe, Germany) was used for the analysis of NMR signals using methanol-*d*_4_ as a solvent. The UV and IR spectra were obtained using Jasco UV-550 (JASCO, Tokyo, Japan) and Perkin–Elmer model LE599 (Perkin–Elmer, Waltham, MA, USA) spectrometer, respectively. ESIMS and HRESI-TOF-MS data were obtained on LCQ Fleet and maXis 4G mass spectrometers (Bruker Daltonics, Bremen, Germany), respectively. Semi-preparative HPLC (Waters, Milford, MA, USA) was performed using a Waters 515 HPLC pump with a 996-photodiode array detector, and Waters Empower software (Pro version 5.0) using a Gemini-NX ODS-column (150 × 10.0 mm and 150 × 21.2 mm). Column chromatography procedures were performed using silica gel (200–400 mesh, Fisher Scientific, Waltham, MA, USA) and Sephadex LH-20 (25–100 µm, Pharmacia Fine Chemical Industries Co., Uppsala, Sweden). Thin-layer chromatography (TLC) was performed using aluminum plates precoated with Kieselgel 60 F_254_ (0.25 mm, Merck, Darmstadt, Germany).

### 3.3. Measurement of Antioxidant and α-Glucosidase Activity

The inhibitory effect on α-glucosidase was measured using α-glucosidase (from *Saccharomyces cerevisiae* (EC 3.2.1.20) [50]. A test sample was mixed with 80 μL enzyme buffer and 10 μL α-glucosidase and incubated for 15 min at 37 °C. Then, after the addition of 10 μL *p*-nitrophenyl α-D-glucopyranoside solution for enzyme reaction, the amount of *p*-nitrophenol that was cleaved by the enzyme was determined by measuring the absorbance at 405 nm in a 96-well microplate reader. Acarbose was used as a positive control. The antioxidant activity was evaluated by measuring the DPPH radical scavenging activity using ascorbic acid as a positive control [50]. The activity (%) was calculated as [100 − (absorbance of sample − blank)/(absorbance of control − blank)].

### 3.4. Quantitation of Phenolic and Flavonoid Contents

The leaves of *E. japonica* were extracted, respectively, with 80% MeOH. The MeOH extract was suspended in H_2_O and partitioned successively with CH_2_Cl_2_, EtOAc, and *n*-BuOH. The total amounts of phenolic and flavonoid contents of the extract and each fraction were quantitated using Folin–Ciocalteu assay and aluminum chloride colorimetric assay, respectively [51].

### 3.5. Extraction and Isolation

For the purification of compounds, the dried powder of *E. japonica* leaves (3.0 kg) was extracted with 80% MeOH (30 L × 2) at room temperature. The MeOH extract (740.0 g) was suspended in H_2_O and partitioned successively with CH_2_Cl_2,_ EtOAc, and *n*-BuOH.

The EtOAc fraction (EJE, 48.5 g) was subjected to MPLC on silica gel eluted with a mixture of CH_2_Cl_2_-MeOH (90:10 to 0:100 gradient) to obtain three subfractions (EJE1-3). Subfraction EJE1 was chromatographed on Sephadex LH-20 eluted with a mixture of *n*-hexane-CH_2_Cl_2_-MeOH (5:5:1) to obtain six subfractions (EJE1A-F). Compounds **12**–**17** and **24** were isolated from EJE1E and EJE1 by semi-preparative HPLC eluted with acetonitrile-H_2_O (43:57) and acetonitrile-H_2_O (13:87), respectively.

The *n*-BuOH fraction (EJB, 138.3 g) was chromatographed on HP-20 eluted with a mixture of H_2_O-MeOH (100:0 to 0:100) to obtain six subfractions (EJB1-6). Subfraction EJB4 was subjected to MPLC on RP-silica gel eluted with a mixture of CH_2_Cl_2_-MeOH (90:10 to 0:100 gradient) to obtain six subfractions (EJB4A-F). Subfraction EJB4B was chromatographed on Sephadex LH-20 eluted with a mixture of CH_2_Cl_2_-MeOH (9:1) to obtain eleven subfractions (EJB4B1-11). Compound **18** was isolated from EJB4B2 by semi-preparative HPLC eluted with acetonitrile-H_2_O (15:85). EJB4B3 was subjected to MPLC on silica gel eluted with a mixture of CH_2_Cl_2_-MeOH (90:10 to 0:100 gradient) to obtain eight subfractions (EJB4B3A-H). Compounds **19** and **30** were purified from EJB4B3C by semi-preparative HPLC eluted with acetonitrile-H_2_O (10:90). Compounds **20** and **21** were isolated from EJB4B3E and EJB4B3G, respectively, by semi-preparative HPLC eluted with acetonitrile-H_2_O (15:85). EJB4B4 was subjected to MPLC on silica gel eluted with a mixture of CH_2_Cl_2_-MeOH (90:10 to 0:100 gradient) to obtain eight subfractions (EJB4B4A-H). Compounds **6**–**7** and **27**–**29** were isolated from EJB4B4C and EJB4B4F, respectively, by semi-preparative HPLC eluted with acetonitrile-H_2_O (15:85). EJB4B5 was subjected to MPLC on silica gel eluted with a mixture of CH_2_Cl_2_-MeOH (90:10 to 0:100 gradient) to obtain eight subfractions (EJB4B5A-H). Compounds **1**–**2** and **3**–**5** were isolated from EJB4B5A and EJB4B5C, respectively, by semi-preparative HPLC eluted with acetonitrile-H_2_O (20:60). Semi-preparative HPLC (acetonitrile-H_2_O, 14:86) of EJB4B5B and EJB4B5H gave compounds **23**, **26** and compounds **22**, **25**, respectively. EJB4B6 was subjected to MPLC on silica gel eluted with a mixture of CH_2_Cl_2_-MeOH (90:10) to obtain six subfractions (EJB4B6A-F). Compounds **9** and **11** were isolated from EJB4B6C and EJB4B6E by semi-preparative HPLC eluted with acetonitrile-H_2_O (14:86), respectively. Semi-preparative HPLC (acetonitrile-H_2_O, 15:85) of EJB4B6F gave compounds **8** and **10**, respectively.

### 3.6. Molecular Docking Studies

Molecular docking studies were performed by using SYBYL-X 2.1.1 (Tripos Ltd., St. Louis, MO, USA) with crystal structures of N-terminal subunit (2QMJ) of human maltase-glucoamylase (MGAM) [52]. The target protein regions were minimized with the Tripos force field and all the water molecules and subunits were removed. The ligand preparation was carried out with the “Sanitize” preparation protocol in SYBYL-X 2.1.1. The binding affinity was expressed in total scores, and the higher the total score, the stronger the binding affinity between protein and ligands. Discovery Studio 2019 Client (Biovia Co., San Diego, CA, USA) was used to visualize the pose of ligand from the docked complex.

## Figures and Tables

**Table 1:** Antioxidant and α-glucosidase inhibitory activity of compounds **1**–**30** from *E. japonica* leaves.

Compound No.	α-Glucosidase Inhibitory Activity (%)	Antioxidant Activity (%)	Compound No.	α-Glucosidase Inhibitory Activity (%)	Antioxidant Activity (%)
1	90.5 ± 4.8	92.3 ± 1.5	16	85.2 ± 8.0	84.4 ± 1.4
2	88.9 ± 3.3	93.3 ± 1.6	17	90.9 ± 8.7	69.6 ± 2.2
3	87.0 ± 9.8	88.4 ± 2.8	18	11.3 ± 2.6	14.4 ± 2.8
4	36.4 ± 9.5	89.4 ± 2.6	19	80.7 ± 4.4	25.0 ± 2.1
5	10.7 ± 7.8	83.6 ± 2.8	20	88.0 ± 5.8	21.5 ± 3.9
6	59.0 ± 3.1	88.6 ± 4.2	21	59.0 ± 2.7	81.0 ± 4.8
7	78.9 ± 3.0	47.3 ± 1.9	22	76.5 ± 4.3	67.4 ± 2.7
8	35.1 ± 5.2	60.6 ± 3.4	23	47.4 ± 2.5	57.2 ± 4.9
9	22.6 ± 8.2	11.6 ± 3.7	24	62.2 ± 3.0	86.0 ± 6.1
10	30.4 ± 3.4	17.9 ± 1.8	25	63.6 ± 3.1	87.6 ± 3.1
11	58.1 ± 5.6	86.2 ± 2.4	26	75.7 ± 9.7	86.0 ± 2.8
12	72.7 ± 2.8	54.4 ± 6.3	27	51.5 ± 8.6	19.2 ± 5.1
13	87.2 ± 9.6	68.2 ± 5.7	28	2.7 ± 7.8	4.8 ± 3.4
14	84.5 ± 2.2	42.6 ± 2.0	29	4.0 ± 4.8	15.5 ± 3.9
15	88.9 ± 8.8	45.1 ± 5.3	30	4.5 ± 2.6	4.7 ± 3.6
PC	53.7 ± 5.9	94.5 ± 4.8			

## Data Availability

The data are contained within the article.

## Supplementary Materials

The following supporting information can be downloaded at https://www.mdpi.com/article/10.3390/molecules30030736/s1, Molecular docking analysis data for compounds **12**–**17**.
