# Supplementary material for: Structural Diversity and Anti-Diabetic Potential of Flavonoids and Phenolic Compounds in Eriobotrya japonica Leaves
Source: Molecules. 2025 Feb 6;30(3):736. doi: 10.3390/molecules30030736 (PMC11820478; doi:10.3390/molecules30030736)

2QMJ (N-terminal subnunit)_SP

Compound	SP docking score (kcal/mol)	H-bond	Pi-Pi stacking	Pi-cation	Water bridge
Co-crystallized ligand	-8.239	ASP203, ASH327, ARG526, ASP542, HIE600			ASN207, TRP406, ASP443
Compound 12	-5.661	ARG202, ASH327, ASP443	TYR299, PHE575		THR205, GLN603
Compound 13	-6.510	GLU404, ASP443, ASP474			
Compound 14	-6.510	GLU404, ASP443, ASP474			
Compound 15	-6.510	GLU404, ASP443, ASP474			
Compound 16	-6.336	ASP203, ASH327, NAG2005			
Compound 17	-7.095	ASH327, GLU404, ASP443, THR544	TYR299		

2QMJ_Co-crystallized ligand (-8.239)

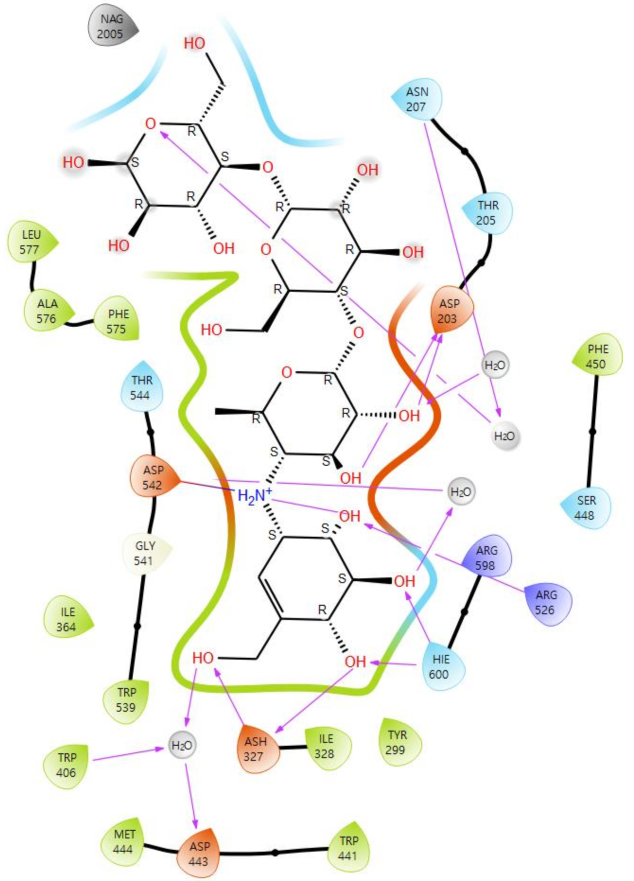

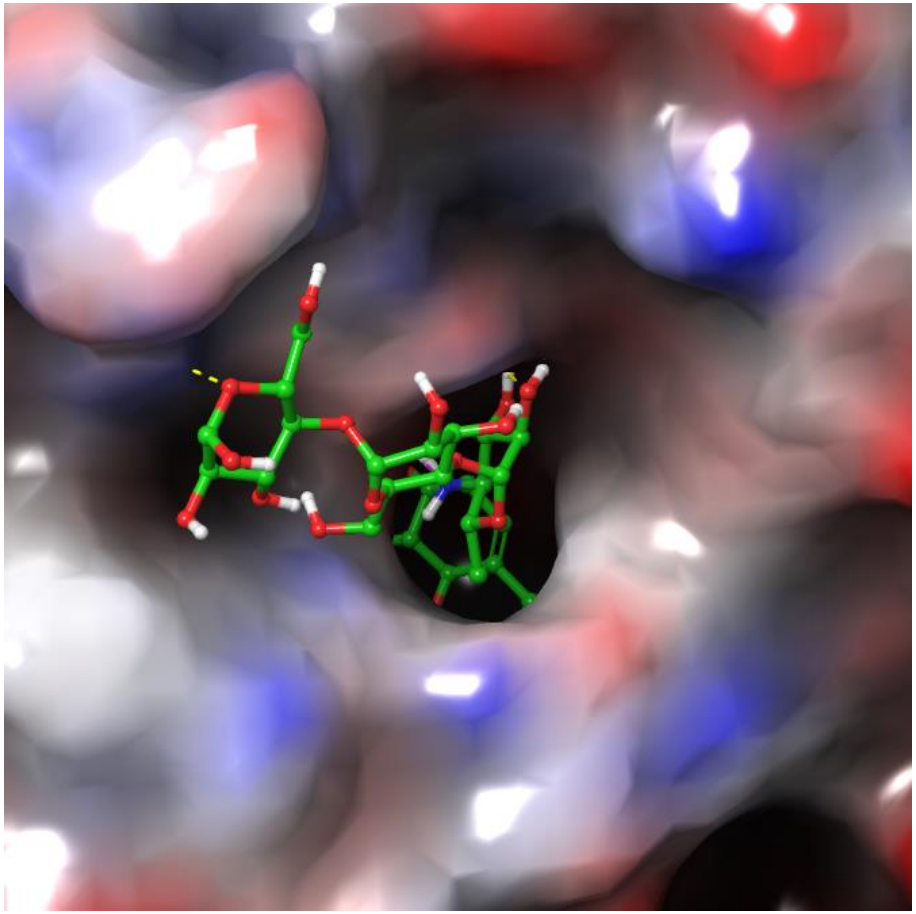

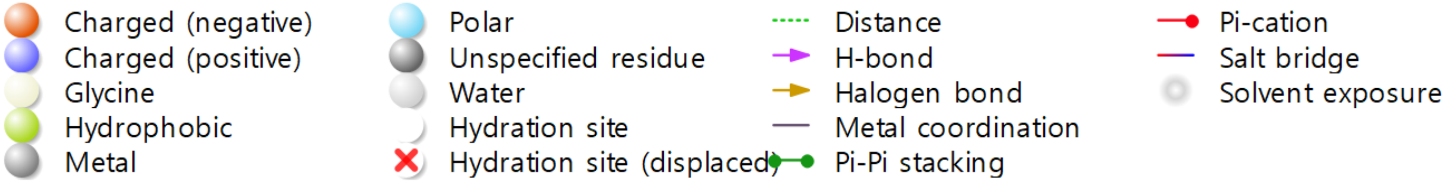

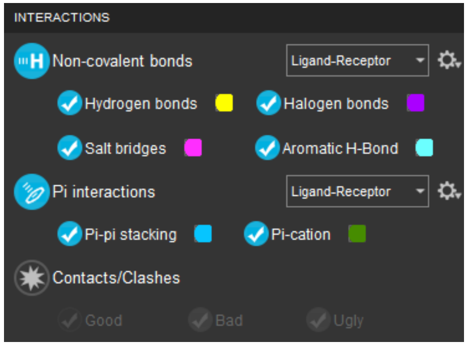

2QMJ_Compound 12 (-5.661)

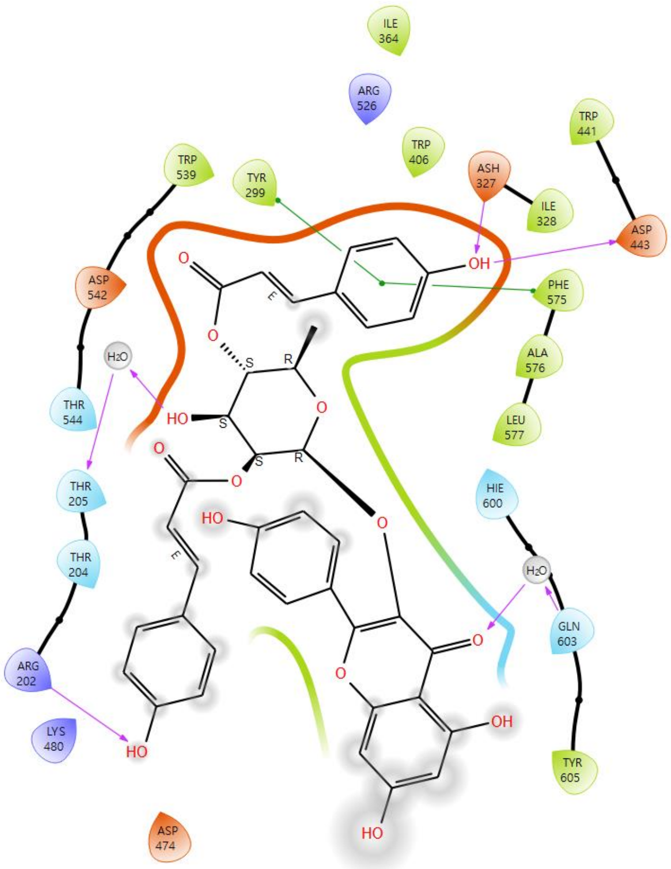

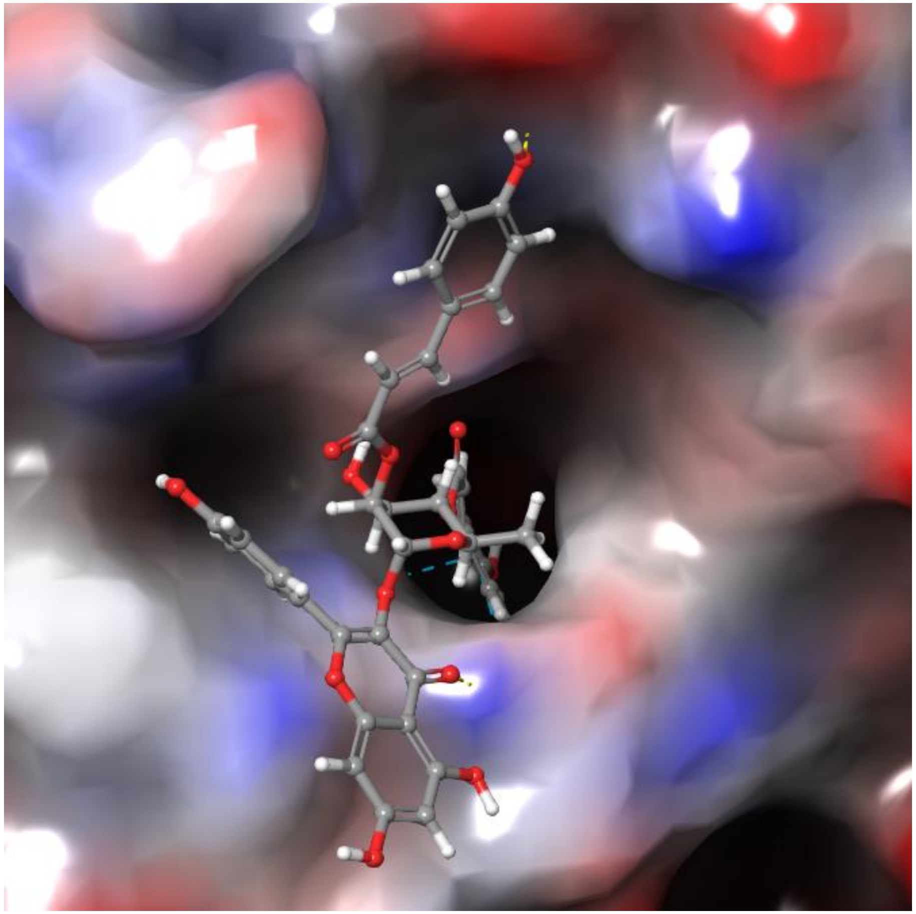

Charged (negative)
Charged (positive)
Glycine
Hydrophobic
Metal
Polar
Unspecified residue
Water
Hydration site
Hydration site (displaced)
Distance
H-bond
Halogen bond
Metal coordination
Pi-Pi stacking
Pi-cation
Salt bridge
Solvent exposure

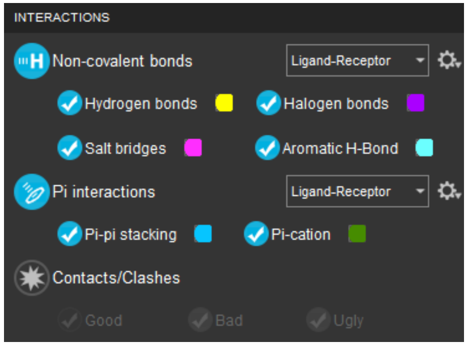

2QMJ_Compound 13~15 (-6.510)

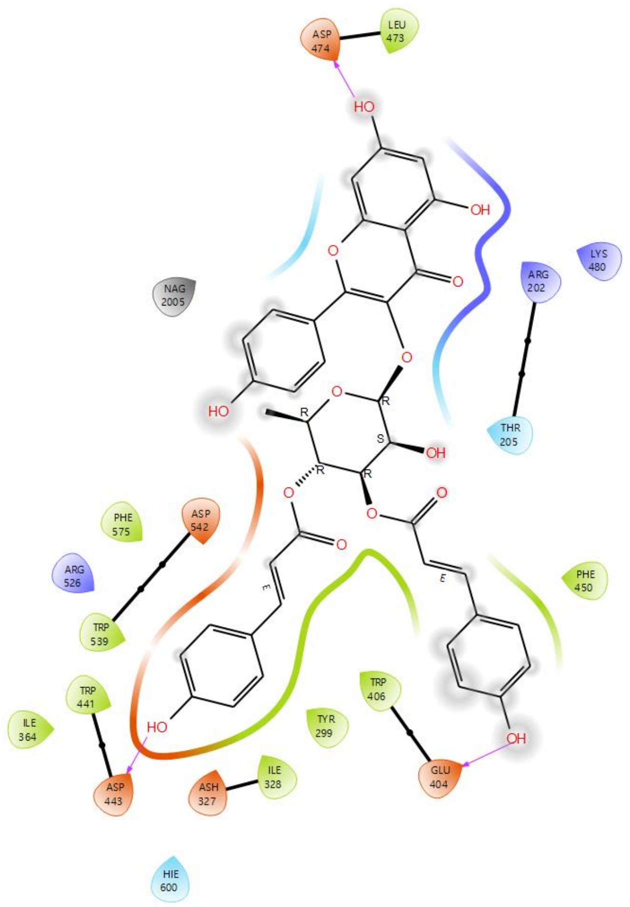

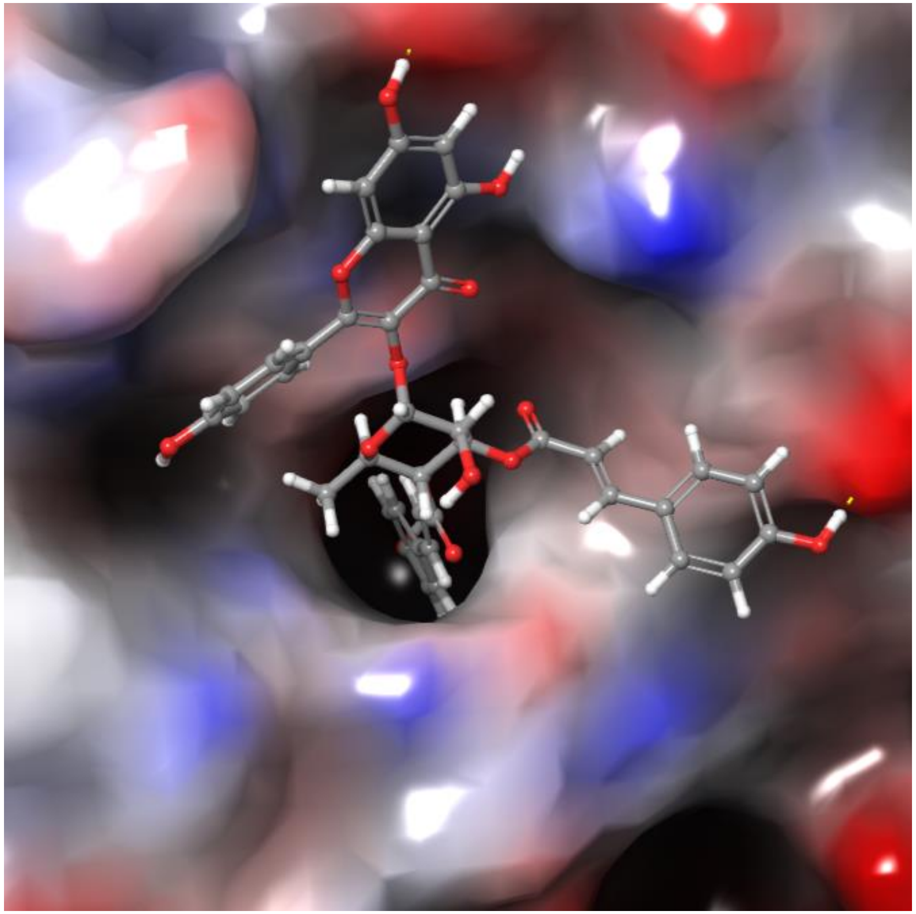

- Charged (negative)
- Charged (positive)
- Glycine
- Hydrophobic
- Metal
- Polar
- Unspecified residue
- Water
- Hydration site
- Hydration site (displaced)
- Distance
- H-bond
- Halogen bond
- Metal coordination
- Pi-Pi stacking
- Pi-cation
- Salt bridge
- Solvent exposure

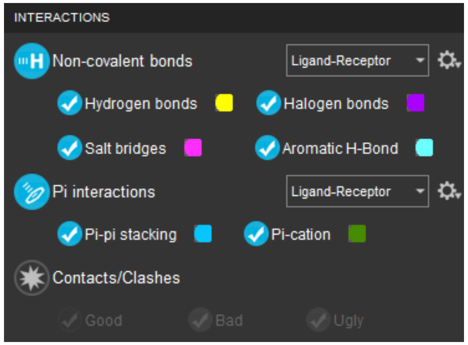

2QMJ_Compound 16 (-6.336)

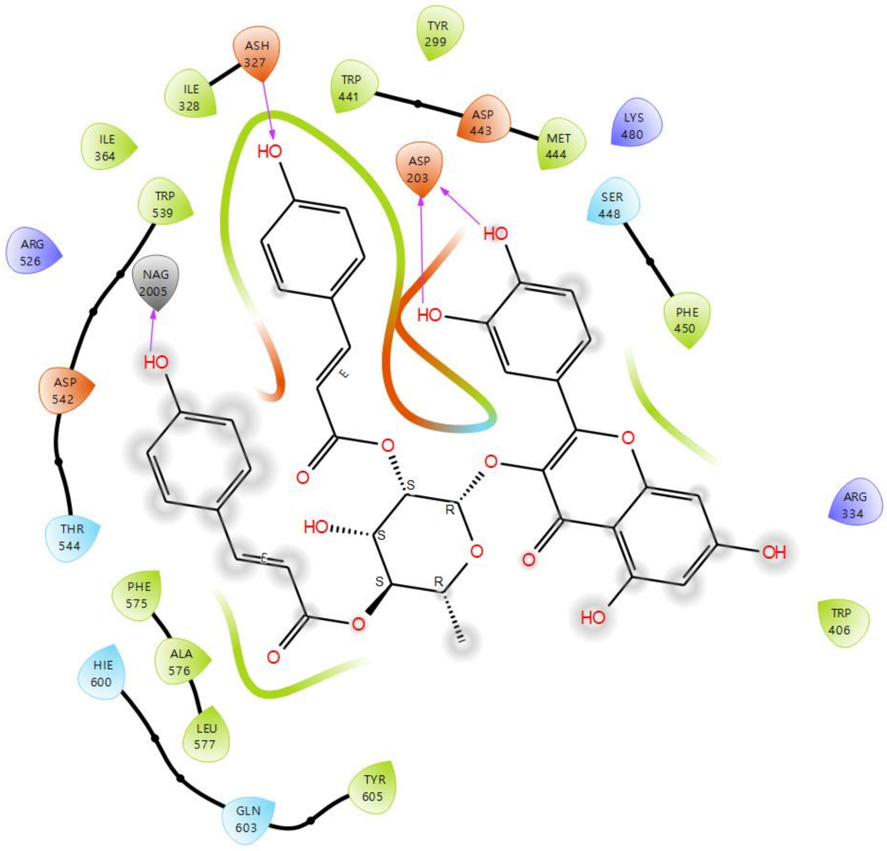

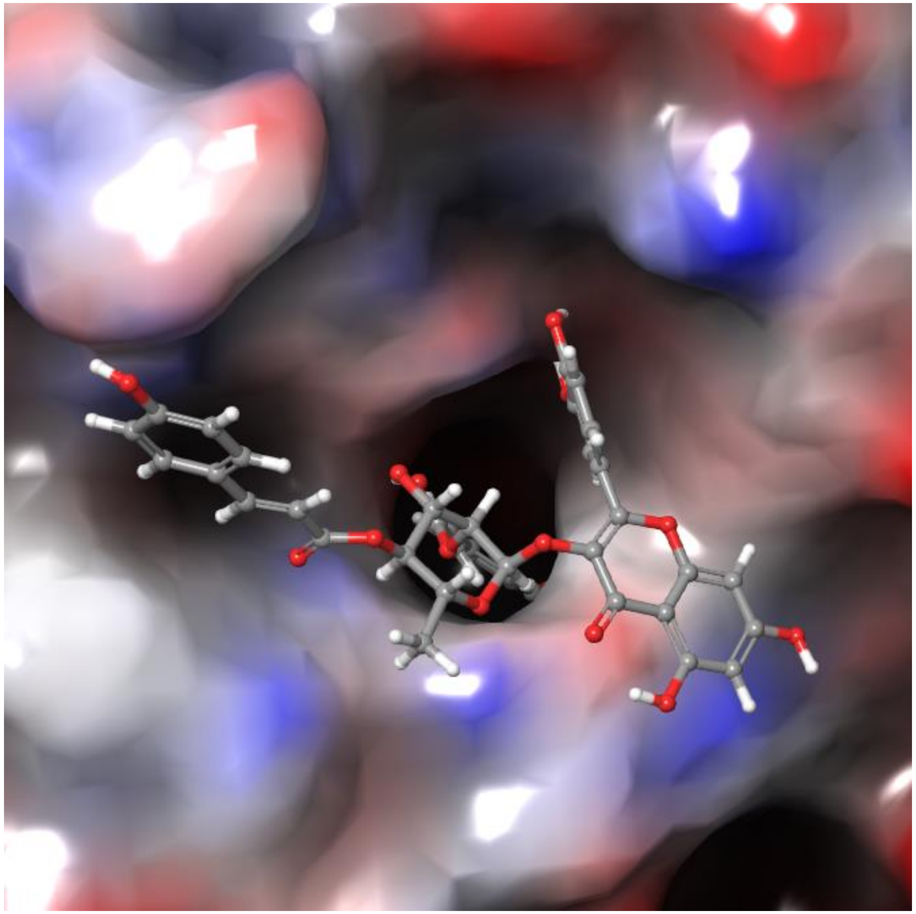

Charged (negative)
Charged (positive)
Glycine
Hydrophobic
Metal
Polar
Unspecified residue
Water
Hydration site
Hydration site (displaced)
Distance
H-bond
Halogen bond
Metal coordination
Pi-Pi stacking
Pi-cation
Salt bridge
Solvent exposure

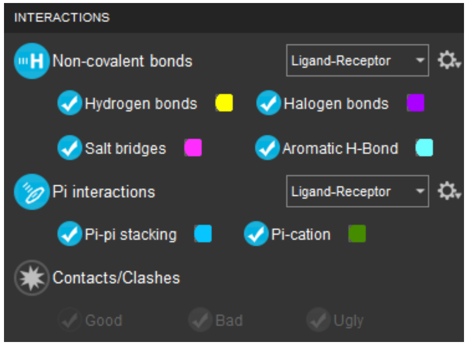

2QMJ_Compound 17 (-7.095)

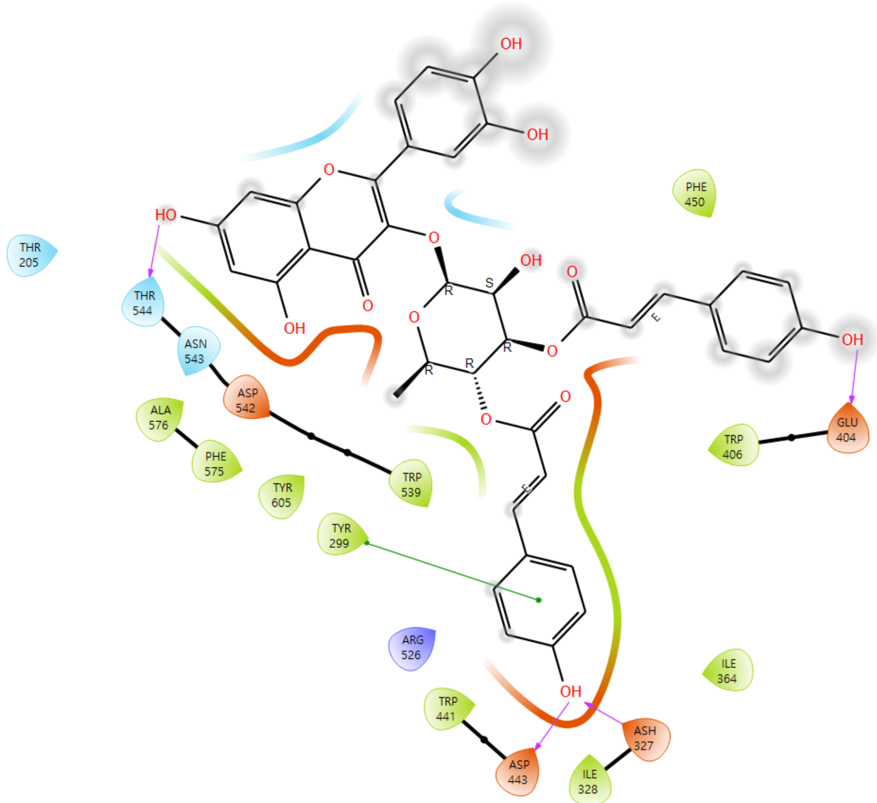

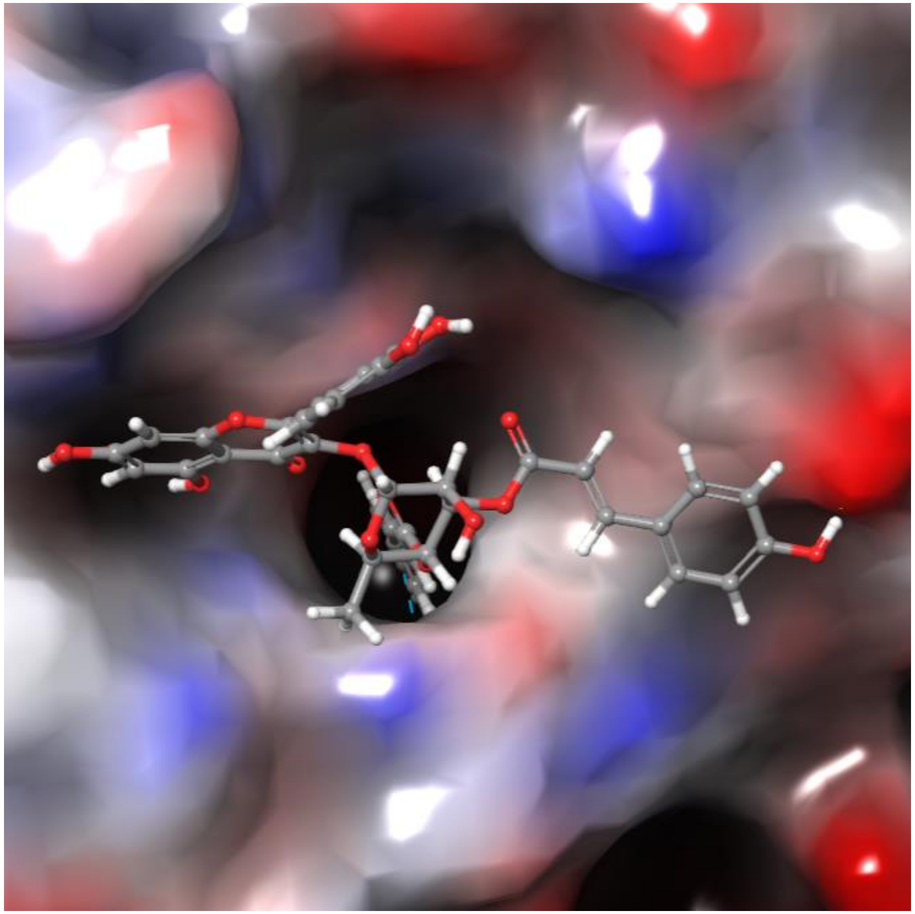

Charged (negative)
Charged (positive)
Glycine
Hydrophobic
Metal

Polar
Unspecified residue
Water
Hydration site
Hydration site (displaced)

Distance
H-bond
Halogen bond
Metal coordination
Pi-Pi stacking

Pi-cation
Salt bridge
Solvent exposure

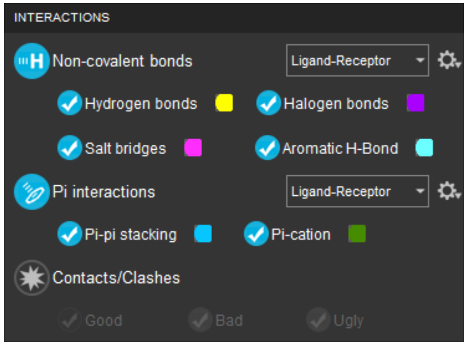

Supplement: Supplementary file 1 [file molecules-30-00736-s001.zip › molecules-3430393-supplementary.pdf]
